# Facile Fabrication of Highly Efficient Chitosan-Based Multifunctional Coating for Cotton Fabrics with Excellent Flame-Retardant and Antibacterial Properties

**DOI:** 10.3390/polym16101409

**Published:** 2024-05-16

**Authors:** Yuan-Yuan Huang, Li-Ping Zhang, Xing Cao, Xin-Yu Tian, Yan-Peng Ni

**Affiliations:** Institute of Functional Textiles and Advanced Materials, Qingdao Key Laboratory of Flame-Retardant Textile Materials, National Engineering Research Center for Advanced Fire-Safety Materials D & A (Shandong), State Key Laboratory of Bio-Fibers and Eco-Textiles, College of Textiles & Clothing, Qingdao University, Qingdao 266071, Chinacaoxingmini1016@163.com (X.C.);

**Keywords:** bio-based, flame retardance, chitosan, cotton fabrics, antibacterial properties

## Abstract

Interest in the development of eco-friendly, sustainable, and convenient bio-based coatings to enhance flame retardancy and antibacterial properties in cotton fabrics is growing. In this work, chitosan was protonated at its amino groups using a method with a high atom economy using an equimolar amount of amino trimethylene phosphonic acid (ATMP), resulting in the fabrication of a single-component chitosan-based multifunctional coating (ATMP-CS), thereby avoiding any additional neutralization or purification steps. Cotton fabrics coated with various loads of ATMP-CS were prepared through a padding–drying–curing process. The morphology, thermal stability, mechanical properties, antibacterial properties, flame-retardant behavior, and flame-retardant mechanism of these fabrics were investigated. The coating exhibited excellent film-forming properties, and it imparted a uniform protective layer onto the surfaces of the cotton fabrics. When the load capacity reached 11.5%, the coated fabrics achieved a limiting oxygen index of 29.7% and successfully passed the VFT test. Moreover, the ATMP-CS coating demonstrated antibacterial rates against *Escherichia coli* and *Staphylococcus aureus* reaching 95.1% and 99.9%, respectively. This work presents a straightforward and gentle approach to fabricating colorless, environmentally friendly, and highly efficient fabric coatings that have potential applications in promoting the use of bio-based materials.

## 1. Introduction

The natural fiber cotton, which is primarily composed of cellulose [1], possesses a diverse range of applications owing to its exceptional comfort, wearability, breathability, biodegradability and warmth retention and moisture absorption properties [2,3]. It not only serves as a clothing material but also finds utility in various daily necessities, thus playing a crucial role in the textile industry. However, cotton fiber has a limiting oxygen index (LOI) of only 18.0%, making it highly susceptible to ignition [4,5]. Once ignited, cotton fabrics quickly spread flames and pose a significant fire hazard. Statistical data highlight the flammability of cotton fabrics as a prominent factor contributing to fires in indoor and public spaces. Moreover, the inherent polysaccharide structure and high hygroscopicity of cotton fibers create an ideal environment for microbial growth that can lead to bacterial proliferation detrimental to fabric performance as well as risks to human health. Consequently, there has been a significant research focus on developing cotton fabrics with exceptional antibacterial properties and flame retardancy [4].

Surface treatment technologies such as impregnation, padding–drying–curing [6,7], the layer-by-layer (LBL) technique [8], gel-sol finishing process [9], and grafting [10,11] are considered the most straightforward and effective methods for imparting flame-retardant or antibacterial properties [12] to cotton fabrics. Building upon these techniques, various functional finishing agents or coating systems have been designed, developed, and applied [13]. Although certain functional flame retardants (e.g., halogenated flame retardants) or antibacterial agents (e.g., triclosan [14]) have demonstrated favorable efficacy, their utilization remains contentious or even prohibited due to their detrimental impact on the environment and human health [15]. The pursuit of efficient, low-toxicity, and environmentally friendly functional finishing agent systems has garnered increasing attention from both academia and industry. Taking the example of flame-retardant cotton fabrics, the environmentally friendly halogen-free flame-retardant systems used at present are primarily based on either a single phosphorus-based flame retardant [12,16] or a synergistic system that combines multiple elements of phosphorus, nitrogen, and silicon [17]. Exemplary representatives include phosphonates, phosphorus/nitrogen-containing siloxanes [18], phosphazenes [5,19], Proban^®^, and Pyrovatex^®^ [20,21] flame-retardant systems. In recent years, bio-based flame retardants [4] have gained popularity due to their non-toxic characteristics, the wide availability of sources of their raw materials, their biodegradability, and their ability to meet safety requirements in a sustainable manner [22,23]. Various biomass materials such as phytic acid [24], chitosan [25], tea polyphenols [26], lignin [27], tannic acid [28,29], and laccase [30] have been investigated for their potential use in developing tailored flame-retardant coating systems specifically designed for cellulose fabrics like cotton. Similarly, the development of antibacterial finishing agents is also progressing toward a path characterized by low toxicity and sustainability, with bio-based antibacterial agents emerging as the preferred choice for the future. Furthermore, multifunctional fabric finishing agents or coatings have emerged as a prominent developmental trajectory.

Chitosan, a positively charged biopolysaccharide, is commonly used as a multifunctional coating [31,32] for fabrics due to its excellent antibacterial properties, carbonization ability, and film-forming properties [33]. However, the flame-retardant effect of coatings containing only chitosan is relatively limited. Therefore, it is often necessary to composite chitosan coatings with other flame retardants (such as LBL systems [34]) or chemically modify [11] them to prepare phosphorylated chitosan in order to achieve the desired flame-retardant effect. Li et al. [27] employed the LBL technique to fabricate a composite coating comprising chitosan and lignosulfonate on the surface of cotton fabric which exhibited remarkable synergistic flame retardancy. With a loading level of 25.2%, the cotton fabric achieved a limiting oxygen index (LOI) value of 26.0%. Li et al. [11] synthesized a N-methylene phosphonic chitosan derivative through the Mannich reaction and applied it to enhance the flame retardancy of cotton fabrics. With a load of 14.2%, the LOI value reached 28.0%, and the material successfully passed the vertical flame test (VFT). Despite its remarkable effectiveness, the LBL method necessitates multiple cycles of soaking, drying, and re-soaking, resulting in overall inefficiency throughout the entire process; the synthesis and purification of phosphorus-containing chitosan derivatives consume a substantial amount of chemical agents.

In recent years, excessive amounts of phosphorus-containing inorganic or organic acids have been used to dissolve chitosan for the preparation of chitosan phosphonate flame retardants or coatings, which have also demonstrated favorable flame-retardant effects on cotton fabrics [32,35]. However, it is worth noting that in almost all previous studies, the amount of phosphorus-containing acid employed far exceeded the required amino protonation. This overuse of phosphorus-containing acid often results in an excessively acidic coating environment due to its low pKa value, thereby inducing the degradation of not only the chitosan itself but also cellulose, consequently compromising fabric properties. Adding more alkali to neutralize excess phosphoric acid can prevent the above-mentioned problems, but it also leads to the wastage of chemicals. Additionally, after applying the coating to fabric, small-molecule phosphate components formed from neutralization still remain on the fabric’s surface. Strictly speaking, not only chitosan derivatives but also excessive phosphonates contribute to flame-retardant effects, which raises doubts about certain research conclusions and calls for attention to the safety of small-molecule phosphates. However, previous researchers seem to have overlooked this issue. Therefore, there is a need for an efficient, environmentally friendly, and easy-to-use chitosan-based multifunctional coating.

In this work, we achieved the protonation and dissolution of chitosan in an aqueous medium by neutralizing its amino groups using an equimolar amount of ATMP, resulting in the successful preparation of a single-component macromolecular chitosan-based coating. The ATMP-CS coating was dipped and finished on cotton fabric through the padding–drying–curing process, and flame-retardant cotton fabric samples with different loads were prepared. We evaluated the thermal stability, flame-retardant behavior, combustion behavior, mechanical properties, antibacterial properties, and flame-retardant mechanism of these fabrics. The fabric achieved a limiting oxygen index of 29.7% and successfully passed the VFT test when its load capacity reached 11.5%. Meanwhile, the antibacterial rates of the ATMP-CS coating against *Escherichia coli* and *Staphylococcus aureus* reached 95.1% and 99.9%, respectively. This work presents a straightforward and gentle approach to constructing colorless, environmentally friendly, and efficient fabric coatings with potential applications in expanding the use of bio-based materials.

## 2. Experiment Section

### 2.1. Material

Chitosan (CS; M_n_ = 200,000; deacetylation degree of 85.0%) was procured from Macklin Biochemical Reagent Co., Ltd. (Shanghai, China). Amino trimethylene phosphonic acid (ATMP; 50 wt.% solution) was purchased from Innochem Technology Co., Ltd. (Beijing, China). Cotton fabric (100%, 170 g/m^2^) was provided by Hong-da Weaving Factory (Dongguan, China). All chemicals were utilized as received without further purification.

### 2.2. Preparation of ATMP-CS Coating

**Figure 1:**
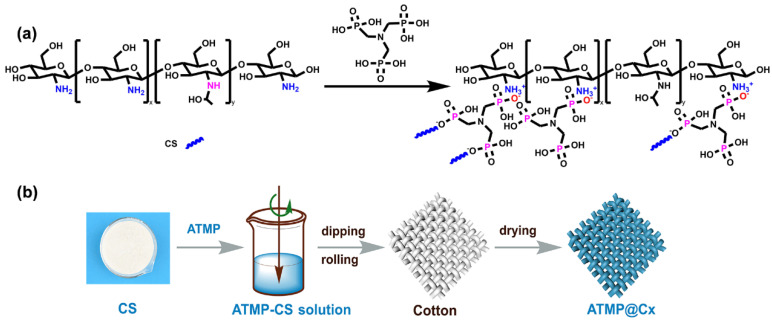
(**a**) Equation of CS and ATMP reaction. (**b**) Schematic diagram of preparation of flame-retardant fabric (ATMP-CS@Cx). The green arrow represents mechanical mixing. Other arrows represent processing processes.

The CS powder and ATMP solution (50.0 wt.%) were added to water in a 1:1 molar ratio of ATMP to CS amino groups, following the principles of an acid–base neutralization reaction (as shown in Figure 1a). The mixture was then stirred at 400 r/min for 3 h to obtain a homogeneous and stable polyelectrolyte solution (the solid content of the CS in the water solution was 1.0 wt.%). 

### 2.3. Preparation of Flame-Retardant Cotton Fabric

Prior to its utilization, the cotton fabric was soaked in a 1.0 mol/L NaOH solution at 100 °C for 1 h, followed by three rinses with deionized water to eliminate residual alkali on the surface, and subsequently dried at 80°C until it reached a constant weight. The preparation process is illustrated in Figure 1b. By calculating the liquid-carrying rate of the fabric, precise control was exercised over its weight gain within the measurement range. The cotton fabric was uniformly coated with the ATMP-CS coating using the dipping–rolling–drying process and then dried at 80°C. To minimize the impact of environmental humidity on fabric weight, the sample was weighed within a 30 s timeframe. The flame-retardant treated cotton fabric is denoted as ATMP-CS@Cx, where “C” represents the cotton fabric and “x” represents the load capacity of ATMP-CS on the cotton fabric (5.5 wt.%, 8.5 wt.%, and 11.5 wt.%).

### 2.4. Characterization

Fourier transform infrared spectroscopy (FT-IR, Nicolet iS50, Thermo-Fisher Scientific, Waltham, MA, USA) and X-ray photoelectron spectroscopy (XPS, ESCALAB Xi+, Thermo-Fisher Scientific) were employed for an analysis of the characteristic peaks and chemical states of the ATMP-CS flame retardants.

The morphology and elemental composition of the coated cotton fabrics and their residues were examined using scanning electron microscopy (SEM, Regulus8100, Hitachi, Tokyo, Japan) and energy-dispersive X-analyzers (EDS, IXRF A500l, IXRF, Austin, TX, USA). The entire surface of the fabric was coated with gold under high vacuum conditions, applying a voltage of 15 kV for a duration of 300 s.

The thermal stability of the coated cotton fabrics was investigated using a thermo-gravimetric analysis (TGA, TGA5500, TA Instruments, New Castle, DE, USA). The experiments were conducted under a nitrogen or air atmosphere at a flow rate of 25 mL/min and a heating rate of 10 °C/min, with the temperature ranging from 40 °C to 700 °C.

A limiting oxygen index instrument (LOI, TTech-GBT2406-4, Tektronix Inc., Beaverton, OR, USA) was utilized to determine the minimum oxygen concentration required for sample combustion (150 mm × 60 mm) based on the GB/T 5454-1997 standard. The vertical flame test (VFT) was conducted using a TTech-GBT2408 vertical burner, and the coated fabrics were prepared in accordance with the GB/T 5455-2014 standard. The dimensions of the samples were 300 mm × 89 mm.

Combustion behavior was investigated using an FTT cone calorimeter in accordance with the ISO 5660-1 standard. The radiation power was set at 35 kW/m^2^, and a fan flow rate of 24 L/s was maintained. The sample dimensions were specified as 100 mm × 100 mm. 

A thermo-gravimetric infrared spectrometer (TG-IR) test was conducted using a PerkinElmer STA6000 thermal analyzer (Perkin-Elmer, Waltham, MA, USA) combined with a Perkin-Elmer Frontier spectrometer(Perkin-Elmer, Waltham, MA, USA), enabling the accurate measurement of the composition, structure, and other properties of the gases released from the samples at various temperatures.

The antibacterial efficacy of the coated cotton fabrics was evaluated according to the GB/T 20944.3-2008 standard, following the experimental protocol described in the referenced literature [34]. The formula is as follows:

Y = (*W*_t_ − *Q*_t_)/*W*_t_ × 100%

Here, *W*_t_ represents the average number of live bacterial colonies in the blank sample after 18 h of oscillation, *Q*_t_ represents the average number of live bacterial colonies in the antibacterial sample, and Y represents the antibacterial rate of the sample, %.

The samples’ mechanical properties were assessed using an INSTRON5967 equipment (Instron, Boston, MA, USA) in accordance with the standard GB/T 3923.1-2013. The samples (200 mm × 50 mm, (area 0.01 m^2^)) were subjected to tensile testing at a crosshead speed of 20 mm/min. 

The air permeability of the samples (40 mm × 50 mm) was determined using a Gellowen Air Permeability Tester (Standard International Group (HK) Limited, Hong Kong, China), following the ISO 9237-1995 standard. 

A whiteness test was conducted using a WSB-V intelligent (Zhejiang TOP Cloud-agriculture Technology Co., LTD., Hangzhou, China.) whiteness tester in accordance with the GB3978-83 standard.

## 3. Results and Discussion

### 3.1. Characterization of ATMP-CS Coating

**Figure 2:**
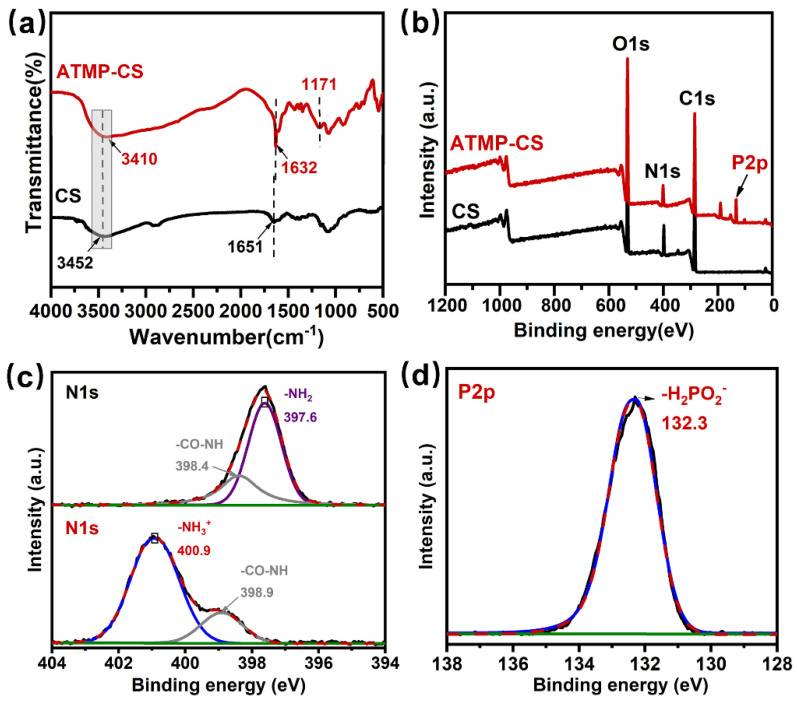
FT-IR spectra of CS and ATMP-CS (**a**). XPS spectra of CS and ATMP-CS (**b**), N1s spectra of CS and ATMP-CS (**c**) and P2p spectra of ATMP-CS (**d**). (The black solid line and red dashed line in (**c**) and (**d**) respectively represent the actual test results and fitting results, while the fitting peaks are denoted by purple, gray, and blue solid lines).

The chemical structure of the ATMP-CS coating was characterized using FTIR and XPS spectra, and the corresponding spectra are shown in Figure 2. In the FT-IR spectrum of CS, the broad absorption band ranging from 3200 cm^−1^ to 3600 cm^−1^ arises due to the superimposition of the stretching vibrations originating from both the O-H and N-H groups. For ATMP-CS, the absorption band exhibits a pronounced red shift which can be attributed to the protonation of the primary amino group leading to the disappearance of its absorption peak, leaving only the -OH group. Moreover, the absorption peak of the bending vibration corresponding to the primary amine group shifts toward the lower band (from 1651 cm^−1^ to 1632 cm^−1^), indicating the successful protonation of -NH_2_ and its transformation into -NH_3_^+^ [35,36]. In addition, the ATMP-CS coating shows a new characteristic absorption peak at 1171 cm^−1^ compared to CS, which can be attributed to the stretching vibration of the associated P=O [37,38,39,40]. XPS spectra were further used to analyze the coating’s elemental composition and chemical states. As shown in Figure 2b, the presence of the element phosphorus was detected in the XPS survey results. From the N1s spectrum of the ATMP-CS coating, it is evident that the characteristic peak at 397.6 eV corresponding to the -NH_2_ group disappears while a new characteristic peak appears at 400.9 eV, indicating the presence of the ammonium ion (-NH_3_^+^)’s peak [41]. Meanwhile, the binding energy of 132.3 eV in the P2p spectrum is also close to that of phosphite. These findings provide further evidence supporting the protonation of the amino group. Finally, the rheological properties of the ATMP-CS solution were analyzed using a rheometer. As shown in Figure S1, the ATMP-CS solution exhibits a low viscosity, facilitating the easy impregnation of fabric and rendering it suitable for the padding–drying–curing process. Moreover, considering the efficiency, simplicity, and effectiveness of the penetration of the coating into the fabric’s interior, we employed a padding–drying–curing process to accomplish the finishing of the ATMP-CS coating onto the fabric.

### 3.2. Morphology and Element Analysis

**Figure 3:**
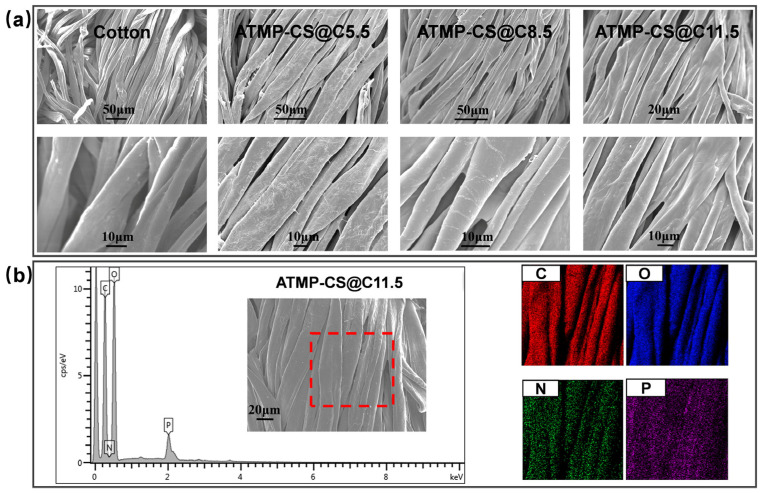
SEM images of cotton and ATMP-CS@Cx fabrics (**a**). Mapping and EDS images for ATMP-CS@C11.5 fabric (**b**) (The output area of the red dotted frame is the EDS scanning range).

SEM-EDS was utilized to analyze the distribution of elements on the cotton fabric surface and its morphology before and after the ATMP-CS coating treatment. As depicted in Figure 3a, the original cotton fabric exhibits a smooth surface with a clear outline of individual fibers and the characteristic texture of natural fibers. By contrast, a thin layer of film adhered to the surfaces of the cotton fibers after they underwent the ATMP-CS coating treatment. At a load of 5.5 wt.%, the coating film is not yet fully continuous, resulting in a relatively rough appearance. As the load increases, each fiber gradually becomes enveloped by an even and seamless film that provided complete coverage while also fostering adhesive connections between adjacent fibers. This also proves that the ATMP-CS coating has good film-forming properties. The EDX images in Figure 3b vividly demonstrate the uniform distribution of C, O, P, and N elements along the fiber direction on the surface of each individual fiber, thereby providing further confirmation of the successful fabrication of a homogeneous flame-retardant coating attached to the fabric surface [8]. It is worth noting that despite the increase in the amount of ATMP-CS coating, the pores between the fibers are still well preserved, ensuring that it does not affect air permeability.

### 3.3. Thermal Stability

**Figure 4:**
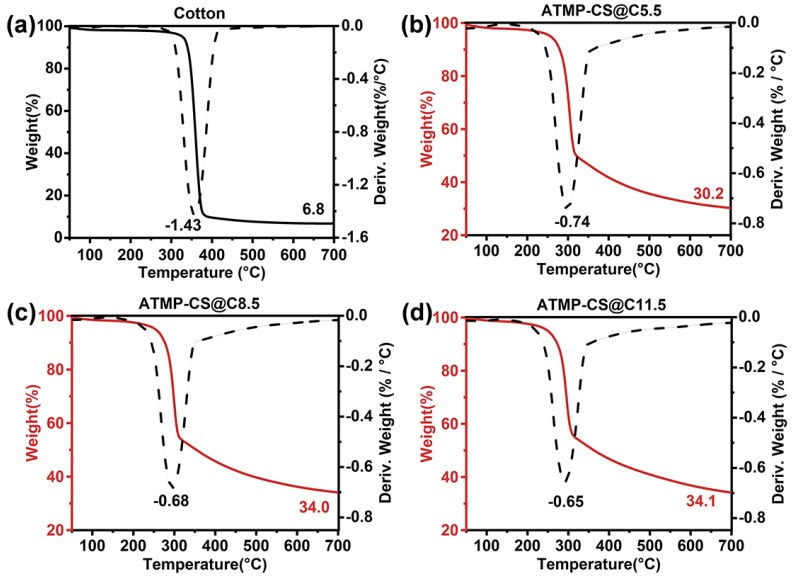
TGA and DTG curves of cotton (**a**), ATMP-CS@C5.5 (**b**), ATMP-CS@C8.5 (**c**) and ATMP-CS@C11.5 (**d**) in nitrogen atmosphere (The solid black line represents the TGA curve of cotton, while the solid red line represents the TGA curve of ATMP-CS@Cx fabrics and the dotted line represents the DTG curve).

TGA tests were conducted under nitrogen and air atmospheres to investigate the thermal stability of the cotton and ATMP-CS@Cx fabrics. Figure 4 illustrates the TGA and DTG curves obtained under a nitrogen atmosphere, with corresponding data listed in Table S1. In all fabric samples under N_2_, the thermal decomposition trend was characterized by a one-step process. The presence of the ATMP-CS coating led to a reduction in both the initial (*T*_5%_) and maximum (*T*_d max_) decomposition temperatures of the fabric, potentially attributed to the catalytic degradation of cellulose and chitosan by phosphite. However, with an increase in the load, there was a significant decrease observed in the maximum decomposition rate (*R*_d max_), indicating a decline in the generation of combustible volatile products. Specifically, the *T*_5%_ of the cotton fabric was 327.4 °C and the *T*_d max_ was 357.4 °C, with an *R*_d max_ of 1.43%/°C. However, for ATMP-CS@C5.5 fabric, the *T*_5%_ decreased to 256.6°C, *T*_d max_ decreased to 299.0 °C, and the *R*_d max_ also decreased to 0.74%/°C. As for the ATMP-CS@C11.5 fabric, all three parameters experienced a further decline to reach 249.7 °C, 290.1 °C, and 0.65%/°C, respectively. Conversely, an increase in load resulted in a significant increase in residual mass, ranging from 6.8 wt.% for cotton to 30.2 wt.% for the ATMP-CS@C5.5 fabric and 34.1 wt.% for the ATMP-CS@C11.5 fabric. Although phosphite played a catalytic role in the initial stage of decomposition, leading to early thermal degradation, the formation of phosphoric acid or polyphosphate structures with high boiling points at elevated temperatures promoted the dehydration and carbonization of chitosan and cellulose, thereby facilitating the retention of more decomposition products in the solid phase. This deduction can be supported by the observed decrease in the *R*_d max_ value and increase in the residual value.

**Figure 5:**
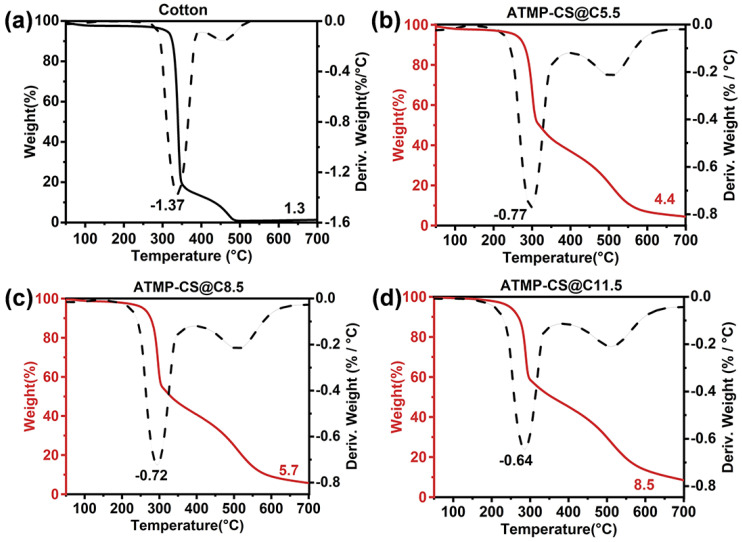
TGA and DTG curves of cotton (**a**), ATMP-CS@C5.5 (**b**), ATMP-CS@C8.5 (**c**) and ATMP-CS@C11.5 (**d**) in air atmosphere (The solid black line represents the TGA curve of cotton, while the solid red line represents the TGA curve of ATMP-CS@Cx fabrics and the dotted line represents the DTG curve).

From the TGA and DTG curves (Figure 5) as well as the corresponding data (Table S2) obtained under the air atmosphere, it is evident that all samples exhibit a typical two-step decomposition process. For the cotton fabrics, the first stage of decomposition primarily occurred at temperatures ranging from 290 °C to 350 °C (with a *T*_d1 max_ value of 338.2 °C) and involved the cracking of glucose units, the breaking of macromolecular chains, and the volatilization of certain substances. The second stage occurred within the temperature range of 400 °C to 480 °C (with a *T*_d2 max_ value of 454.7 °C) as the residue underwent further oxidation reaction with atmospheric oxygen, leading to subsequent degradation. For the ATMP-CS@Cx fabrics, both *T*_5%_ and *T*_d1 max_ also decreased with an increasing load, which is attributed to the accelerated early decomposition facilitated by the ATMP-CS coating. Similar to the case of nitrogen, the decomposition induced by ATMP-CS facilitated dehydration and carbonization, resulting in a greater retention of decomposition products within the solid phase. As a result, there was a significant reduction in the rate of mass loss, as evidenced by a significant decrease in *R*_d1 max_. This process facilitated the formation of more stable residuals, thereby enhancing their resistance to oxidative degradation and resulting in a shift of *T*_d2 max_ toward higher temperatures. Ultimately, the residual mass of the ATMP-CS@Cx fabrics in the air atmosphere was also significantly enhanced.

### 3.4. Flame Retardant Performance

**Figure 6:**
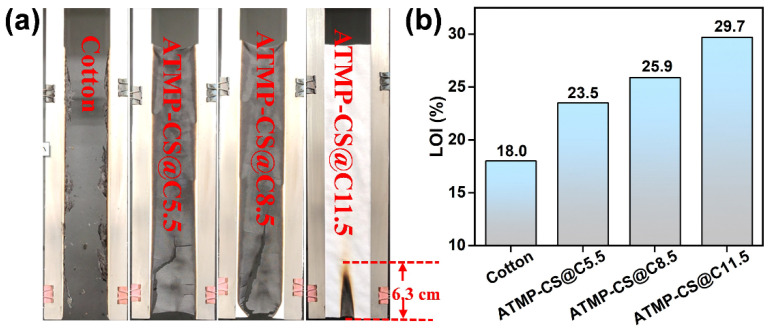
Video capture of cotton fabric and ATMP-CS@Cx fabrics during vertical burning test (**a**) and LOI (**b**).

The VFT and LOI are pivotal parameters for evaluating the flammability of textiles as their results can reflect a fabric’s resistance to ignition and its propensity to sustain combustion post ignition. Figure 6 and Table 1 present specific VFT and LOI test results. The cotton fabric was highly combustible, with an LOI value of a mere 18.0%. During the VFT test, it rapidly burned out upon ignition, exhibiting an after-flame time of 20 s and an after-glow time of 24 s and leaving behind negligible residue. After undergoing the ATMP-CS coating treatment, the flame retardancy of the fabric was obviously improved. Specifically, as the coating load increased, there was a significant rise in the LOI value of the ATMP-CS@Cx fabric from the 18.0% of cotton to 23.5% for ATMP-CS@C5.5, further increasing to 25.9% for ATMP-CS@C8.5 and reaching 29.7% for ATMP-CS@C11.5. During the VFT test, the ATMP-CS@Cx fabric exhibited a pronounced self-extinguishing phenomenon during the VFT test, leading to reductions in the after-flame time, after-glow time, and damage length. The ATMP-CS@C5.5 fabric, despite not passing the VFT test, exhibited a reduced ignition time of 10 s and no smoldering. Additionally, it did not completely burn out but maintained “its fabric shape”, thereby indicating the protective efficacy of the coating on the fabric. When the load capacity was further increased to 11.5%, the ATMP-CS@C11.5 fabric was able to successfully pass the VFT test without any afterburning or smoldering phenomena, and the damage char length value was also reduced to 6.3 cm. 

**Figure 7:**
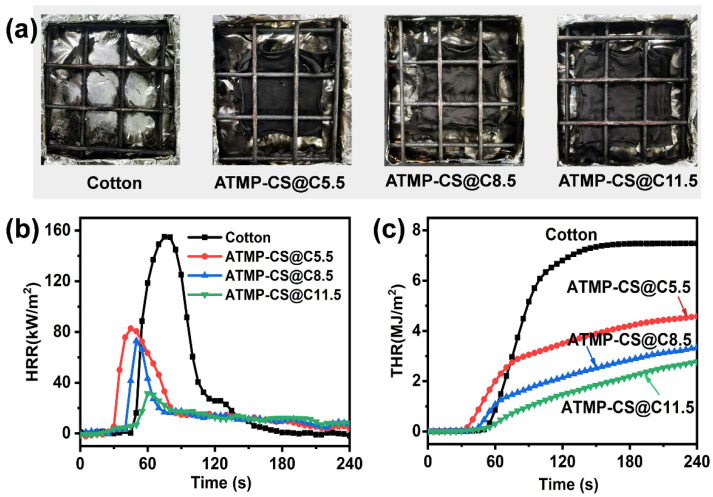
Digital photos for cotton and ATMP-CS@Cx samples after CCT test (**a**). HRR curves (**b**) and THR curves (**c**).

A cone calorimetry test (CCT) can assess the combustion behavior of materials under specific conditions and provide a plethora of parameters, including the ignition time (TTI), peak heat release rate (pHRR), total heat release rate (THR), total smoke release (TSR), average heat release rate (Av-HRR), fire spread index (FIGRA), and mass loss rate. Table 2 presents specific result data after a CCT test, while Figure 7 illustrates HRR and THR curves, along with digital images of char residues after the CCT tests. After undergoing the ATMP-CS coating treatment, the heat releases of fabrics were significantly suppressed. 

As depicted in Figure 7, compared to cotton fabric, the pHRR values of the ATMP-CS@Cx fabrics decrease from 154.9 kW/m^2^ to 82.8 kW/m^2^ for the ATMP-CS@C5.5 fabric and further decline to 73.0 kW/m^2^ for the ATMP-CS@C8.5 fabric, ultimately achieving a remarkable reduction of 85.5% down to 22.4 kW/m^2^ for the ATMP-CS@C11.5 fabric. The THR values of the ATMP-CS@Cx fabrics exhibit a similar trend, with the ATMP-CS@C11.5 fabric demonstrating a THR value of 2.6 MJ/m^2^, indicating a significant reduction of 65.3% compared to that of cotton fabric (7.5 MJ/m^2^). Furthermore, the ATMP-CS@Cx fabrics exhibit significantly lower Av-HRR, FIGRA, and mass loss rate values compared to pure cotton fabric, thereby indicating decelerated combustion propagation and a reduced fire hazard. As for smoke release behavior, the TSP value of cotton fabric itself is negligible, and there is no significant change in the TSP value before and after coating treatment. It is noteworthy that the TTI values of the ATMP-CS@Cx fabrics exhibit an initial shift to an earlier time followed by a subsequent delay. This phenomenon can be attributed to the promotion of the early decomposition of cellulose and chitosan by the ATMP, resulting in an earlier TTI. Simultaneously, this early decomposition facilitates the formation of a protective char layer, and as the load increases, it further enhances the carbonization effect, rendering the fabric less susceptible to ignition. The formation of a protective carbon layer on the fabric surface effectively hinders the transfer of heat, oxygen, and combustible gases, thereby resulting in an increased residual mass. As depicted in Figure 7a, the cotton fabric is almost entirely consumed, with only minimal residue remaining; however, the ATMP-CS@Cx fabrics maintain their “approximate shape” remarkably well, with a residual mass of up to 77.2%. The aforementioned test results demonstrate that the application of the ATMP-CS coating can significantly enhance the flame retardancy of cotton fabric.

### 3.5. Flame-Retardant Mechanism Analysis

**Figure 8:**
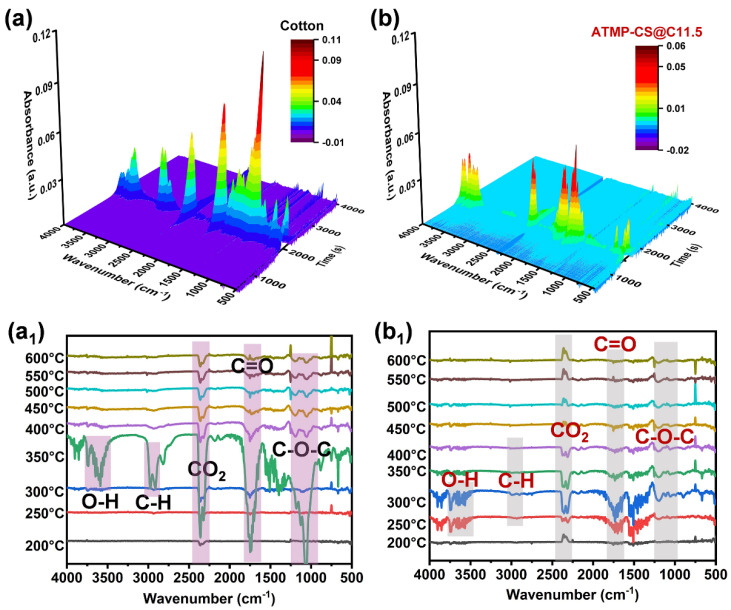
Three-dimensional images (**a**,**b**) of gas phase products of cotton and ATMP-CS@C11.5 samples during thermal degradation and IR spectra at different temperatures (**a_1_**,**b_1_**).

To further explore the flame-retardant mechanism of the ATMP-CS coating on cotton fabric, a TG-IR test was conducted to analyze the volatile products released during the thermal decomposition process of the untreated and treated cotton fabrics. Figure 8 presents the FT-IR spectra of volatile products obtained from the cotton fabric and ATMP-CS@C11.5 fabric throughout the thermal decomposition process, including 3D spectrograms and infrared spectra collected at various temperatures. The infrared spectra of both samples exhibit characteristic bands originating from the decomposition of polysaccharides, including hydroxyl compounds (3400–3700 cm^−1^) [19], hydrocarbons (2800–3200 cm^−1^) [42], carbonyl compound (1600–1850 cm^−1^) [43,44], CO_2_ (2250–2400 cm^−1^), and ether compounds (950–1250 cm^−1^) [42,45]. In contrast, the absorption peaks of the volatile products of coated fabrics were observed at lower temperatures, aligning with the TGA results and providing further confirmation of early decomposition. Furthermore, a significant reduction in the intensity of each peak can be clearly observed for the ATMP-CS@C11.5 fabric, particularly for hydrocarbon compounds and ether compounds, indicating a notable decrease in the release of flammable gaseous products compared to the pure cotton fabric. The remarkable decrease in flammable gaseous products is also in line with the conjecture that ATMP facilitates charring.

**Figure 9:**
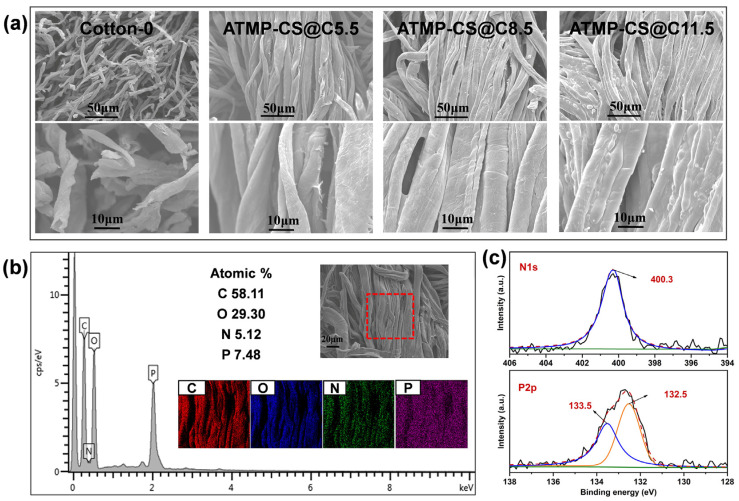
SEM images of char residue from cotton and ATMP-CS@Cx fabrics (**a**). EDS mapping images of char residue on ATMP-CS@C11.5 fabric (**b**). N1s and P2p spectra of char residue on ATMP-CS@C11.5 fabric (**c**) (The black solid line and red dashed line in (**c**) represent the actual test results and fitting results, while the fitting peaks are denoted by blue and orange solid lines).

SEM-EDS and XPS techniques were further employed to analyze the surface morphology and chemical composition of the char residues. From the SEM image of residual ash in the cotton fabric, it is evident that the fabric structure was completely destroyed, with fibers fracturing and giving rise to a hollow and voluminous carbon residue. In contrast, even after the CCT test, ATMP-CS@Cx fabrics maintained their fabric structural integrity and pristine fiber continuity impeccably, appearing devoid of any fractures. Furthermore, a bubbling layer of expanded carbon can be distinctly observed on the surfaces of the residual fibers; it becomes increasingly prominent as the load augments. Additionally, as depicted in Figure 9b, the elements C, N, O, and P exhibit a distinctive fibrous distribution pattern [46], suggesting that these four elements are uniformly distributed on the surface of the ATMP-CS@Cx fabrics. The continuous film layer formed by the ATMP-CS coating, enveloping each individual fiber, facilitates prompt carbonization and exhibits a remarkable expansion flame-retardant effect. As a result, it leads to the formation of a dense carbon layer that effectively isolates combustible gases from the surrounding air and heat, thereby impeding further combustion reactions to protect the fibers. The chemical composition of the ATMP-CS@C11.5 fabric carbon residue after the CCT test was detected using XPS technology. Figure 9c shows the XPS spectrum. The presence of C, N, O and P elements in the carbon residue can be observed from the overall spectrum. The N1s spectra showed that nitrogen occurred mainly in the form of pyridine nitrogen (400.3 eV). The P2p spectra show that the binding energy of 133.5 eV corresponds to the characteristic peak of metaphosphate. The results show that nitrogen and phosphorus are involved in the process of carbonization and the formation of a carbon layer on the coated fabric. The results of an analysis of gas-phase decomposition products and residues indicate that the ATMP-CS coating predominantly contributes to the flame-retardant effect in the condensed phase.

### 3.6. Antibacterial Performance

**Figure 10:**
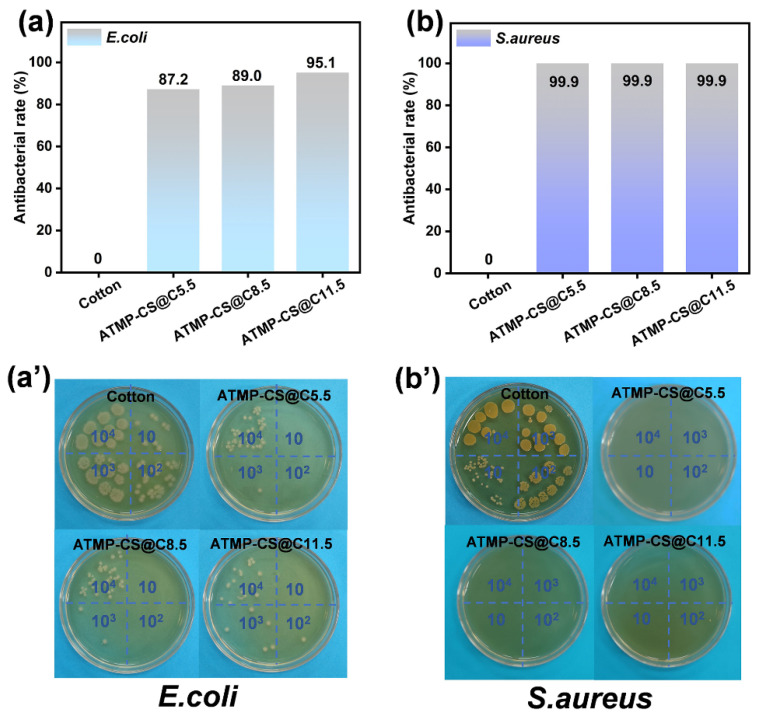
Antibacterial rates of cotton and ATMP-CS@Cx samples (**a**,**b**) and petri dish photos (**a’**,**b’**).

The antimicrobial efficacy of the cotton and ATMP-CS@Cx fabrics against *Escherichia coli* (*E. coli*) and *Staphylococcus aureus* (*S. aureus*), representing Gram-negative and Gram-positive bacteria, respectively, was evaluated using the oscillating method according to GB/T 20944.3-2008. As shown in Figure 10, the number of residual bacterial colonies on the culture dish containing the ATMP-CS@Cx fabric was significantly lower than that on the culture dish containing the cotton fabric. Among them, the antibacterial rates of all the ATMP-CS@Cx fabrics against *S. aureus* can reach 99.9%, demonstrating the excellent efficacy of the ATMP-CS coating in combating Gram-positive bacteria [47]. Meanwhile, the antibacterial rates of the ATMP-CS@C5.5 fabric, ATMP-CS@C8.5 fabric, and ATMP-CS@C11.5 fabric against *E. coli* were 87.5%, 89.0%, and 95.1%, respectively, indicating that the ATMP-CS coating can also effectively enhance the antibacterial effect of cotton fabric against Gram-negative bacteria; however, a certain threshold must be reached for a more significant antibacterial effect to occur. The antibacterial mechanism of protonated chitosan can be divided into two aspects: first, the positive charge carried by the -NH_3_^+^ in CS can be adhere to the cell surface and destroy the cell wall; second, cations that infiltrate into cells absorb anionic substances within them and disrupt normal physiological activities, ultimately leading to bacterial destruction [26].

### 3.7. Whiteness, Air Permeability, and Mechanical Property

**Figure 11:**
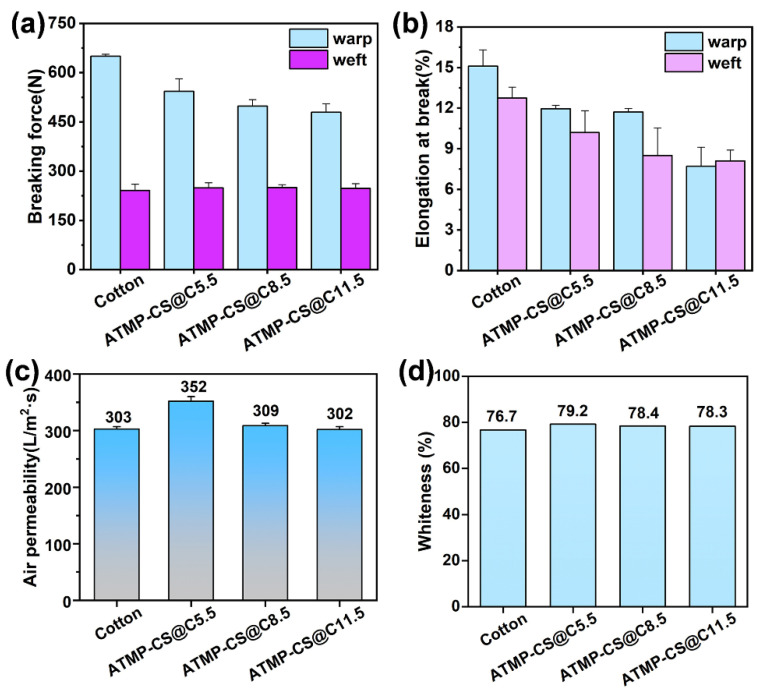
Mechanical property (**a**,**b**), air permeability (**c**), and whiteness (**d**) of cotton and ATMP-CS@Cx fabrics.

The effect of the ATMP-CS coating on the mechanical properties of the fabrics was evaluated by determining the fracture force in both the warp and weft directions before and after undergoing the coating treatment. As shown in Figure 11a, the maximum warp and weft breaking forces at tensile fracture of cotton fabric are 650 N and 241 N, respectively. Compared with cotton fabric, the ATMP-CS@Cx fabrics exhibit higher weft-breaking force, displaying a trend of first increasing and then decreasing with an increasing coating load, while, in the warp direction, their breaking force gradually decreases as the load increases. The reason for this may be that the warp tension in cotton fabric is greater during the weaving process, which damages the structure of the fabric. When the fabric breaks, the ratio of the measured maximum tensile length to the original length is the elongation at break. It can be seen from Figure 11b that the elongation at break of cotton fabric decreases in a certain range. This indicates that the ATMP-CS coating coated the fiber, resulting in a decrease in the toughness of the fabric. 

The air permeability of fabric not only affects its wear performance but also significantly influences its industrial applicability. Figure 11c illustrates the results of the air permeability test conducted on the ATMP-CS@Cx fabrics. It is observed that the air permeability of the treated fabrics was not adversely affected, and even a minimal amount of ATMP-CS coating can enhance the air permeability. Specifically, in comparison to cotton (303 L/m^2^·s), the air permeability of the ATMP-CS@C5.5 fabric exhibits a significant improvement of 16.2%, reaching a value of 352 L/m^2^·s. As for the ATMP-CS@C11.5 fabric, the air permeability falls back to 302 L/m^2^·s, which is comparable to that of cotton. Whiteness serves as another important indicator directly impacting the fabrics’ aesthetics and quality level. As shown in Figure 11d, the whiteness of the samples slightly increased from 76.7% (untreated cotton) to 79.2% (ATMP-CS@C5.5), 78.4% (ATMP-CS@C8.5), and 78.3% (ATMP-CS@C11.5). The above results suggest that the ATMP coating, owing to its gentle characteristics, will not result substantially harm the physical properties of the fabric during processing.

## 4. Conclusions

In this study, chitosan was protonated at its amino groups using a method with high atomic economy using an equimolar amount of ATMP, resulting in the fabrication of a chitosan-based flame-retardant and antibacterial multifunctional coating for cotton fabric that is facile, environmentally friendly, and highly efficient. Throughout the coating preparation and application process, there is no requirement for the excessive introduction of chemical reagents as it consists solely of a single macromolecular component. Consequently, this procedure ensures cleanliness without causing substantial fabric damage or leaving behind residual small-molecule byproducts. The coating exhibits excellent film-forming properties, and through immersion and a padding–drying–curing treatment, it imparts a uniform protective layer onto the surface of cotton fabric. At a coating load of 11.5%, the coated cotton fabric successfully passed the VFT test, and the LOI value reached 29.7%. In addition, the antibacterial rates of the ATMP-CS coating against *Escherichia coli* and *Staphylococcus aureus* reached 95.1% and 99.9%, respectively. Importantly, this colorless and eco-friendly coating had no damaging effect on the whiteness and breathability of the fabrics. This study provides a promising strategy for the eco-friendly and efficient synthesis of bio-based flame retardants, as well as the development of a colorless, environmentally friendly, and effective flame-retardant coating for fabrics.

## Figures and Tables

**Table 1:** Data from vertical burning test and LOI values.

Samples	Add-on (%)	After-Flame Time (s)	After-Glow Time (s)	Damaged Length (cm)	LOI (%)
Cotton	0	20.0	24.0	30.0	18.0
ATMP-CS@C5.5	5.5	10.0	0	30.0	23.5
ATMP-CS@C8.5	8.5	8.0	0	30.0	25.9
ATMP-CS@C11.5	11.5	0	0	6.3	29.7

**Table 2:** CCT data for cotton and ATMP-CS@Cx fabrics.

Samples	TTI (s)	pHRR (kW/m^2^)	THR (MJ/m^2^)	TSR (m^2^)	Av-HRR (kW/m^2^)	FIGRA (kW/(m^2^.s))	Residues (%)
Cotton	42.5 ± 2.1	146.6 ± 11.8	7.4 ± 0.5	0.04 ± 0.01	29.1 ± 0.4	2.0 ± 0.2	1.7 ± 0.2
ATMP-CS@C5.5	24.0 ± 2.8	87.1 ± 6.1	4.9 ± 0.2	0.02 ± 0.001	17.9 ± 0.6	1.8 ± 0.008	16.2 ± 1.2
ATMP-CS@C8.5	37.5 ± 0.7	74.4 ± 2.0	3.9 ± 0.3	0.09 ± 0.02	14.8 ± 1.1	1.4 ± 0.06	28.7 ± 0.9
ATMP-CS@C11.5	47.0 ± 1.5	19.3 ± 4.5	2.8 ± 0.3	0.02 ± 0.002	10.6 ± 0.6	0.2 ± 0.08	77.2 ± 2.0

## Data Availability

The original contributions presented in this study are included in the article/Supplementary Material, further inquiries can be directed to the corresponding author.

## Supplementary Materials

The following supporting information can be downloaded at https://www.mdpi.com/article/10.3390/polym16101409/s1, Figure S1: Viscosity curve of ATMP solution with shear rate; Table S1: TGA and DTG dates of cotton and ATMP-CS@Cx fabrics in nitrogen atmosphere; Table S2: TGA and DTG dates of cotton and ATMP-CS@Cx fabrics in air atmosphere; Table S3: The properties data of the fabric itself.
