# Supplementary material for: Facile Fabrication of Highly Efficient Chitosan-Based Multifunctional Coating for Cotton Fabrics with Excellent Flame-Retardant and Antibacterial Properties
Source: Polymers (Basel). 2024 May 16;16(10):1409. doi: 10.3390/polym16101409 (PMC11125217; doi:10.3390/polym16101409)
Supplement: Supplementary file 1 [file polymers-16-01409-s001.zip › polymers-2990253-supplementary.pdf]

Facile fabrication of highly efficient chitosan-based multifunctional coating for cotton fabrics with excellent flame retardant and antibacterial properties

Yuan-Yuan Huang , Li-Ping Zhang , Xing Cao , Xin-Yu Tian , Yan-Peng Ni *

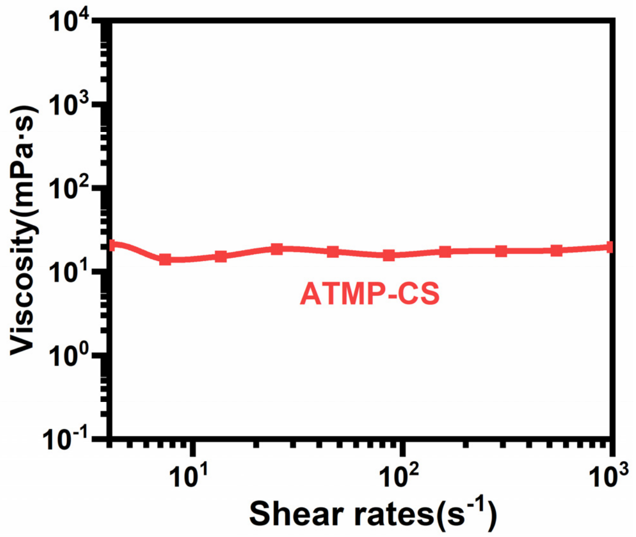

Figure S1. Viscosity curve of ATMP solution with shear rate

Table S1. TGA and DTG dates of cotton and ATMP-CS@Cx fabrics in nitrogen atmosphere.

Samples	Nitrogen			
	$T_{5\%}$(°C)	$T_{d\,max}$(°C)	$R_{d\,max}$(%/°C)	Residues(700°C, wt.%)
Cotton	327.4	357.4	1.43	6.8
ATMP-CS@C5.5	256.6	299.0	0.74	30.2
ATMP-CS@C8.5	254.2	294.9	0.68	34.0
ATMP-CS@C11.5	249.7	290.1	0.65	34.1

Table S2. TGA and DTG dates of cotton and ATMP-CS@Cx fabrics in air atmosphere.

Samples	Air					
	$T_{5\%}$ (°C)	$T_{d1\ max}$ (°C)	$R_{d1\ max}$ (%/°C)	$T_{d2\ max}$ (°C)	$R_{d2\ max}$ (%/°C)	Residue (700°C, wt.%)
Cotton	309.9	338.2	1.37	454.7	0.15	1.3
ATMP-CS@C5.5	255.9	298.3	0.77	505.1	0.21	4.4
ATMP-CS@C8.5	257.1	293.7	0.72	508.8	0.21	5.7
ATMP-CS@C11.5	247.5	285.7	0.64	510.0	0.21	8.5

Table S3. The properties data of the fabric itself

Samples	Breaking force(N)		Elongation at break(%)		Air permeability (L/m^2·s)	Whiteness (%)
	Warp	Weft	Warp	Weft		
Cotton	650	241	15.1	12.7	303	76.7
ATMP-CS@C5.5	543	243	12.0	10.2	352	79.2
ATMP-CS@C8.5	498	254	11.7	8.5	309	78.4
ATMP-CS@C11.5	479	241	7.7	8.1	302	78.3
